# A WD40-Repeat Protein From the Recretohalophyte *Limonium bicolor* Enhances Trichome Formation and Salt Tolerance in *Arabidopsis*

**DOI:** 10.3389/fpls.2019.01456

**Published:** 2019-11-12

**Authors:** Fang Yuan, Bingying Leng, Haonan Zhang, Xi Wang, Guoliang Han, Baoshan Wang

**Affiliations:** ^1^Shandong Provincial Key Laboratory of Plant Stress, College of Life Sciences, Shandong Normal University, Ji’nan, China; ^2^Maize Research Institute, Shandong Academy of Agricultural Sciences, Jinan, China

**Keywords:** *Arabidopsis*, heterologous expression, *Limonium bicolor*, root hair, salt stress, trichome, WD40-repeat protein

## Abstract

The *Arabidopsis thaliana* WD40-repeat protein *TRANSPARENT TESTA GLABRA1* (*TTG1*) controls epidermis development, playing opposite roles in trichome differentiation and root hair formation. We isolated and characterized *LbTTG1* (encoding a WD40-repeat protein with high sequence similarity to TTG1) from the recretohalophyte *Limonium bicolor*, which actively excretes absorbed salt *via* a salt gland. The complete open reading frame of *LbTTG1* was 1,095 bp, encoding a protein of 364 amino acids, and showed highest expression during the salt gland initiation stage. We heterologously expressed *LbTTG1* in wild type and *ttg1-13 Arabidopsis* plants to verify the protein’s function, and the copies of LbTTG1 were identified in transgenic strains using southern blotting. Trichomes were extremely induced on the first true leaves of plants heterologously expressing *LbTTG1*, whereas no trichomes were produced by *ttg1-13* plants. Conversely, plants heterologously expressing *LbTTG1* produced fewer root hairs than *ttg1-13* plants. In plants heterologously expressing LbTTG1 compared to controls, epidermis differentiation genes (*GLABRA1* and *GLABRA3*) were up-regulated while genes encoding negative regulators of trichome development (*TRIPTYCHON* and *CAPRICE*) were down-regulated. Under increased NaCl concentrations, both of the transgenic lines showed enhanced germination and root length, and accumulated less malondialdehyde (MDA) and Na^+^ and produced more proline, soluble sugar, and higher glutathione S-transferase activity, compared with the *ttg1-13* mutant. These results indicate that LbTTG1 participates in epidermis development in *Arabidopsis*, similarly to other WD40-repeat proteins, and specifically increases salt tolerance of transgenic *Arabidopsis* by reducing ion accumulation and increasing osmolyte levels.

## Introduction

Soil salinization inhibits the growth and production of crops worldwide. Currently, 800 million hectares of land is affected by elevated salinity, and poorly designed irrigation approaches are causing rapid expansion of saline farmland worldwide (Munns and Tester, 2008). Finding ways to make use of large areas of saline land is urgent as a means to expand available arable land (Song et al., 2017). Few crops can survive in salt-affected regions, which leads to substantially reduced production and often results in soil degradation and desertification (Flowers and Colmer, 2008; Song and Wang, 2015). Thus, it is necessary to improve the salt tolerance of non-halophytic plants by transforming them with genes conferring salt tolerance (Yuan et al., 2015).

Halophytes that survive and complete their life cycles in environments with ≥200 mM NaCl can provide a broad bank of genes for improving the salt tolerance of non-halophytes. Two key points of focus should be identifying suitable halophytes and choosing key salt-tolerance genes. A highly efficient *Agrobacterium*-mediated transformation system has been established for *Limonium bicolor*, a recretohalophyte that can actively excrete absorbed salt to the outside *via* a salt gland (Ding et al., 2010; Deng et al., 2015). Salt glands are specific and visible epidermal structures that make recretohalophytes distinct from all non-halophytes and other types of halophytes. A large number of mutants involved in salt gland development and salt secretion were screened by an efficient autofluorescence method (Yuan et al., 2013), and the distribution patterns of salt glands have been demonstrated (Leng et al., 2018). Five distinct stages of epidermis differentiation have been discerned in leaves (Yuan et al., 2015), and the ultrastructures of salt glands have also been observed (Feng et al., 2014; Feng et al., 2015). Given this existing foundation, *L. bicolor* represents a good model plant for studying salt tolerance and development of the salt gland.

Our previous studies illustrated a series of genes that may participate in salt gland development (Yuan et al., 2015) and salt secretion (Yuan et al., 2016). Surprisingly, genes reported to be involved in trichome initiation differentiation are found in *L. bicolor*, such as those with homology to *GLABRA1* (*GL1*), *TRANSPARENT TESTA GLABRA1* (*TTG*), *GLABRA3* (*GL3*), *TRIPTYCHON* (*TRY*), and *CAPRICE* (*CPC*) (Yuan et al., 2015). Given that no trichomes are observed in *L. bicolor* (Leng et al., 2018), we speculate that salt glands of *L. bicolor* may evolve from a trichome-like structure under the control of similar regulatory genes.

In *Arabidopsis*, trichomes act as a physical barrier against biotic stress, and their differentiation network has been clearly illustrated. TTG1, encoding a WD40-repeat protein, participates in trichome initiation, flavonoid production, seed coat mucilage production, and seed oil and storage protein accumulation, and negatively regulates root hair development (Walker et al., 1999). AtTTG1 and AtGL1 combine with AtGL3 or ENHANCER OF GLABRA3 (AtEGL3) to form a TTG1-GL3/EGL3-GL1 complex, which directly regulates AtGL2 and AtTTG2 in the positive regulation of trichome initiation (Zhao et al., 2008). Negative regulators of trichome development are mainly MYB proteins, including TRY, CPC, ENHANCER OF TRY AND CPC1 (ETC1), and ETC2 (Kirik et al., 2004; Tominaga-Wada and Nukumizu, 2012; Tominaga-Wada and Wada, 2016). The functions of homologous genes associated with trichome development in other species have been verified using heterologous expression in *Arabidopsis*. For example, the transgenic expression of *CsTTG1* in cucumber (*Cucumis sativa*) and *Arabidopsis* enhances trichome number (Chen et al., 2016), and the cucumber *CsGL1* gene mutant *csgl1* shows abnormal trichomes on leaves, stems, flowers, and fruits, with expression of papillae instead (Li et al., 2015; Zhao et al., 2015). Therefore, heterologous expression is a useful tool for investigating gene function.

Here, we identified a gene encoding a WD40-repeat protein with high sequence similarity to TTG1 of *Arabidopsis* by comparing transcriptome data of *L. bicolor* (Yuan et al., 2015) with expression data for all homologous genes involved in trichome differentiation. WD40-repeat proteins (also known as WD or beta-transducin repeats) are short ∼40 amino acid motifs, often terminating in a Trp-Asp (W-D) dipeptide, and are involved in negative regulation of root hairs and positive of trichomes (Payne et al., 2000; Zhang et al., 2003). This gene, named *LbTTG1* in *L. bicolor*, may play an important role in salt gland development due to its high expression during salt gland differentiation. *LbTTG1* played an important role in epidermis formation and can enhance the salt tolerance of *Arabidopsis*, and the possible reason was also illustrated.

## Materials and Methods

### Plant Materials and Growth Conditions

Seeds of *L. bicolor* were collected from a saline, inland environment (N37°20′; E118°36′) in the Yellow River Delta, Shandong, China. Dry seeds were stored in a refrigerator at 4°C for 6 months before use. Seeds were surface-sterilized in 70% ethanol for 5 min, followed by 6% (v/v) sodium hypochlorite (239305, Sigma, USA) with vigorous shaking for 15–20 min, and then washed thoroughly with sterile distilled water before being germinated on Murashige and Skoog (1962) (MS) basal medium containing 3% (w/v) sucrose and 0.9% (w/v) agar, adjusted to pH 5.8 with KOH before autoclaving. Seeds were cultured at 28 ± 3°C/23 ± 3°C (day/night) at a light intensity of 600 µmol/m^2^/s (15-h photoperiod) and 70% relative humidity. The first true leaves were separately collected at different leaf developmental stages—stage A (undifferentiation, 4–5 days after sowing), stage B (salt gland differentiation, 6–7 days), stage C (stomata differentiation, 8–10 days), stage D (epidermis differentiation, 11–16 days), and stage E (mature, more than 17 days)—for RT-PCR and gene cloning according to (Yuan et al., 2015).

The *Arabidopsis thaliana* ecotype Col-0 (Columbia-0) was used as a control. The homozygous *Arabidopsis* AT5G24520 mutant *ttg1-13* (CS67772), obtained by fast neutron mutagenesis, was ordered from the *Arabidopsis* Biological Resource Center. Seeds of Col-0 and CS67772 were sterilized three times using 75% ethanol for 3 min and then three times using 95% ethanol for 1 min, and washed five times with distilled water. Seeds were sown on sterile half-strength (1/2) MS medium containing 0.8% (w/v) sucrose and 0.8% (w/v) agar (pH 5.8) for germination. After 2 days of vernalization at 4°C, seeds were cultured at 22°C/18°C (day/night) under a 16 h/8 h light/dark cycle with a light level of 150 µmol/m^2^/s and 70% relative humidity (Sui et al., 2017). After culture for 1 week, seedlings were transplanted to pots (10 cm in diameter and 8 cm in height) containing well-mixed soil (soil: vermiculite: perlite, 3:1:1) for further flowering and transformation.

### Full-Length Clone of *LbTTG1* From *L. bicolor*

According to (Yuan et al., 2015), approximately 5,000 leaves from stage A, 4,000 from stage B, 2,000 from stage C, 1,000 from stage D, and 1,000 from stage E were separately dissected at the same time of day (8:00 a.m. to 10:00 a.m.), and all leaves were immediately frozen in liquid nitrogen and stored –80°C until use. Total RNA from different samples was extracted using a Total Plant RNA Extraction kit (Karroten, Beijing, China) and purified using ethanol and 3 mol/L sodium acetate to precipitate polysaccharides. Purified total RNA was quantified using a NanoDrop ND-2000 spectrophotometer (NanoDrop products, Wilmington, DE, USA); the concentration of total RNA was greater than 700 ng/µl with optical density (OD)_260/280_ 2.0–2.2 and OD_260/230_ ≥ 1.0. After electrophoresis to measure integrity, cDNA was obtained using a ReverTra Ace^®^ quantitative PCR (qPCR) RT kit (TOYOBO Co., Ltd, Japan) according to the manufacturer’s instructions.

Lb106501, homologous to the *Arabidopsis* gene AT5G24520 (*AtTTG1*) and named *LbTTG1*, was identified after mapping the leaf developmental transcriptome of *L. bicolor* to the Uniref database (http://www.uniprot.org/) by applying Blastx with an E-value threshold of 0.005 (Yuan et al., 2015). The assembled sequence of *LbTTG1*, as a reference for the full-length clone, was obtained from previous transcriptome results (Yuan et al., 2015). All primers were designed using Primer Premier 5.0 (**Table S1**). A specific intermediate fragment of *LbTTG1* was first obtained using cDNA of stage A leaves as template with primers *LbTTG1*-S and *LbTTG1*-A; primers for 3′ RACE amplification were designed according to this intermediate fragment. cDNA for 3′ RACE amplification was reverse transcribed using QT primer instead of the primer mix. The 3′ RACE sequence was obtained after continuous 3′-end amplification using primer Q0 paired with *LbTTG1*3′-GSP1 and primer Q1 paired with *LbTTG1*3′-GSP2. The 5′-end sequence of *LbTTG1* was obtained by 5′ RACE using a 5′ RACE kit (5′ RACE System for Rapid Amplification of cDNA Ends, Version 2.0, Invitrogen) with primers *LbTTG1*5′-GSP1 and *LbTTG1*5′-GSP2. The full-length sequence of *LbTTG1* was determined by combining the sequencing results, and the full-length cDNA of *LbTTG1* was obtained using primers *LbTTG1* CDS-S and *LbTTG1* CDS-A.

### Bioinformatics Analysis of *LbTTG1* in *L. bicolor*

The conserved domain of LbTTG1 was predicted using the online tool SMART (http://smart.embl-heidelberg.de/). A phylogenetic tree was reconstructed using the neighbor-joining method with MEGA5.1 software (www.megasoftware.net) and ClustalX software, with statistical support for nodes obtained from at least 1,000 trials.

### Subcellular Localization of LbTTG1 by Transient Expression

The open reading frame (ORF) region of *LbTTG1* was cloned into the pCAMBIA1300 vector, containing a CaMV 35S promoter, hygromycin resistance gene, and GFP reporter gene, by double digestion with *Kpn*I and *BamH*I after TA cloning using pEASY-T3 vectors with primers *LbTTG1* OE-S and *LbTTG1* OE-A (**Table S1**). The resulting p1300-LbTTG1 vector was transformed into onion epidermis cells using *Agrobacterium tumefaciens* strain GV3101 (Sun et al., 2007). Fluorescence signals of labeled *LbTTG1* were detected by microscopy (TCS S8 MP two-photon laser scanning confocal microscope, Leica, Germany).

### qPCR Analysis at Different Developmental Stages

Total RNAs of A-E stage leaves, stems, roots, flowers, and mature leaves under 200 mM NaCl treatment for 24 h were extracted for real-time PCR. qPCR primers were designed using Beacon Designer™ Free Edition software (version 7.8). The amplification procedure consisted of 94°C for 5 min, followed by 35 cycles of 30 s at 94°C, 1 min at 51°C, and 1 min at 72°C. SYBR Green qPCR was carried out in a fluorometric thermal cycler (Bio-Rad CFX96^™^ Realtime PCR System) using SYBR Premix Ex Taq^™^ (TaKaRa, Mountain View, CA) in 20 µl reaction mixtures containing 10 µl of 2×SYBR Premix Ex Taq™ (TaKaRa), 50 ng cDNA, and 0.2 µM each primer (**Table S1**, *Lbtubulin* RT and *LbTTG1* RT) using *Lbtubulin* as an internal control. The PCR thermal cycle was as follows: denaturation at 95°C for 5 min and 40 cycles at 94°C for 20 s, 58°C for 15 s, and 65°C for 15 s. Three replicate biological experiments were performed. Relative expression levels were calculated using the formula 2^–ΔΔC(T)^.

### Vector Construction and Transformation of *Arabidopsis* Col-0 and *ttg1-13*

The *LbTTG1* ORF was cloned into the pCAMBIA3301 vector under the control of the CaMV 35S promoter to generate p35S::LbTTG1, using primers *LbTTG1* OEAt-S and LbTTG1 OEAt-A (**Table S1**) according to the instructions of the In-Fusion^®^ HD Cloning kit (Clontech Laboratories, Inc.). The p35S::LbTTG1 vector was introduced into *A. tumefaciens* strain EHA105, which was then transformed into *A. thaliana* Col-0 and *ttg1-13* by the *Agrobacterium*-mediated floral dip method (Clough and Bent, 2010). After screening with herbicide for three consecutive generations, homozygous *Col* 35S::*LbTTG1* and *ttg* 35S::*LbTTG1* lines were retained for qPCR.

Plants heterologously expressing *LbTTG1* were identified by PCR using primers *LbTTG1*V-S and *LbTTG1*V-A on genomic DNA. Total RNA of several strains of *Col* 35S::*LbTTG1* and *ttg* 35S::*LbTTG1* was then extracted using a FastPure Plant Total RNA Isolation kit (Vazyme) according to the manufacturer’s instructions. qPCR was conducted to evaluate the expression level of *LbTTG1* in *Col* 35S::*LbTTG1* and *ttg* 35S::*LbTTG1* using *LbTTG1*RT-S and *LbTTG1*RT-A primers. Amplification of the *actin* gene of *Arabidopsis* was used as an internal control (primers *Atactin*RT-S and *Atactin*RT-A). Three replications were carried out for each transgenic line. Three lines with high, medium, and low *LbTTG1* expression levels, respectively, of *Col* 35S::*LbTTG1* and *ttg* 35S::*LbTTG1* were used for further experiments.

### Southern Hybridization of *LbTTG1* in *L. Bicolor* and Transgenic Lines of *Arabidopsis*

Genomic DNA was extracted from 2-week-old seedlings of *L. bicolor* and transgenic lines of *Arabidopsis* using cetyltrimethylammonium bromide (CTAB) method (Guo et al., 2015), then 10 µg was digested with *Hind*III. After electrophoresis in a 0.7% agarose gel at 25 V and 4°C overnight, positive control (pGBKT7-LbTTG1 digested with *Nde*I to obtain LbTTG1) and all samples were transferred to a HyBond N^+^ membrane (Amersham, UK) using upward capillary method for 20 h. Digested DNA was fixed to the membrane at 80°C for 2 h. The probe was synthesized by primers *LbTTG1*P-S and *LbTTG1*P-A (**Table S1**), and was labeled with Digoxigenin-11-dUTP (PCR DIG Probe Synthesis Kit, Roche). DNA gel blot hybridization was performed by Zoonbio Biotechnology Co., Ltd (Nanjing, China) using DIG DNA labeling and detection kit (Roche). After pre-hybridization at 37°C for 2 h and probe denaturation, hybridization was incubated at 37°C overnight. Membrane was washed two times using 2×SSC/0.1%SDS for 5 min at room temperature, then twice for 15 min in 1×SSC/0.1%SDS buffer at 65°C. After blocking for 30 min, the membrane was immersed in antibody solution (Anti-Digoxigenin-AP, Roche) for 30 min. Hybridized membrane was detected in the presence of CSPD (C_18_H_20_ClNa_2_O_7_P), and the hybridized signals were visualized on X-ray film after 1 h.

### Phenotypic Observation of Trichome and Root Hair Development in *Col* 35S::*LbTTG1* and *ttg* 35S::*LbTTG1*

Phenotypes of transgenic plants were observed in the T_3_ generation, and photographs of 1-week-old homozygous seedlings of the T_3_ generation were taken under a dissecting microscope (Nikon, Japan). The number of trichomes on the first true leaf was calculated for 10 plants of each line. Root hair length and number were also examined in 5-day-old *Col* 35S::*LbTTG1* and *ttg* 35S::*LbTTG1* seedlings; three replicates of 20 plants from each line were performed. The same root position (region from 2 to 3 cm from the root tip) was chosen for calculating root hair number and length. The numbers of trichomes and root hairs were calculated using ImageJ software (http://rsb.info.nih.gov/ij/).

### Effect of NaCl Concentration on Salt Tolerance of Different Transgenic Lines: Percentage Germination

Three *Col* 35S::*LbTTG1* and three *ttg* 35S::*LbTTG1* lines with high, medium, and low LbTTG1 expression levels, respectively, were used for salt treatment. All seeds were sowed on 1/2 MS medium with different NaCl concentrations (0, 50, 100, and 150 mM). After 24 h, seed germination percentage was calculated based on radicles breaking the seed coats for more than 1 mm. Germination percentage (%) = number of germinated seeds/total number of seeds × 100%. Fifty seeds of each line were sowed in each treatment, and three replicates were performed.

### Effect of NaCl Concentration on Salt Tolerance of Different Transgenic Lines: Root Length and Physiological Indicators

All seeds were germinated on 1/2 MS medium for 24 h before uniform seedlings were transplanted to medium with different NaCl concentrations (0, 50, 100, and 150 mM) to observe the effect of NaCl on root growth. Five-day-old seedlings were photographed for root length measurement using ImageJ software. Twenty seedlings were measured for each line.

Four-day-old, uniform seedlings grown on 1/2 MS medium were transplanted into soil for gradient NaCl treatment (0, 50, 100, and 150 mM). Two-week-old seedlings under different NaCl treatments were pooled to 0.5 g for the measurement of the contents of Na^+^ and K^+^, MDA, proline, and soluble sugar according to (Han et al., 2019) and (Guo et al., 2018). Ion concentrations were determined using a flame photometer (M410, Sherwood, UK). Glutathione S-transferase (GST) activity was determined according to (Dinler et al., 2014) that the formation of the conjugate1-chloro-2,4-dinitrobenzene (CDNB) using reduced glutathione resulted in the increase of absorbance at 340 nm. Five replicates were performed for each line.

### RT-qPCR of Trichome Formation and Stress-Related Genes

Five-day-old seedlings of all lines grown on 1/2 MS medium were collected for RNA extraction. RT-qPCR was performed using primers targeting genes related to trichome differentiation, including *AtTTG1*, *AtGL1*, *AtGL3*, *AtEGL3*, *AtCPC*, and *AtTRY*, listed as genename-S and genename-A (e.g. *AtTTG1*-S and *AtTTG1*-A) in **Table S1**.

Two-week-old seedlings of all lines grown on 1/2 MS medium were transferred to 1/2 MS liquid medium supplied with 100 mM NaCl solution for 0 and 3 h. Seven stress-related marker genes were selected for RT-qPCR: *AtSOS1*, *AtSOS2*, *AtSOS3*, *AtP5CS1*, *AtP5CS2*, *AtGSTU5*, and *AtAREB1* (**Table S1**). Three replicate biological experiments were performed. Relative expression levels were calculated using the formula 2^–ΔΔC(T)^. *Atactin* (*Atactin*RT-S and *Atactin*RT-A) was used as an internal control.

### Statistical Analysis

Statistical analysis was performed using SPSS at *P* = 0.05 (Duncan’s multiple range tests). ANOVA with orthogonal contrasts and mean comparison procedures was used to detect differences between treatments.

## Results

### *LbTTG1* Encoded WD40-Repeat Protein

**Figure 1:**
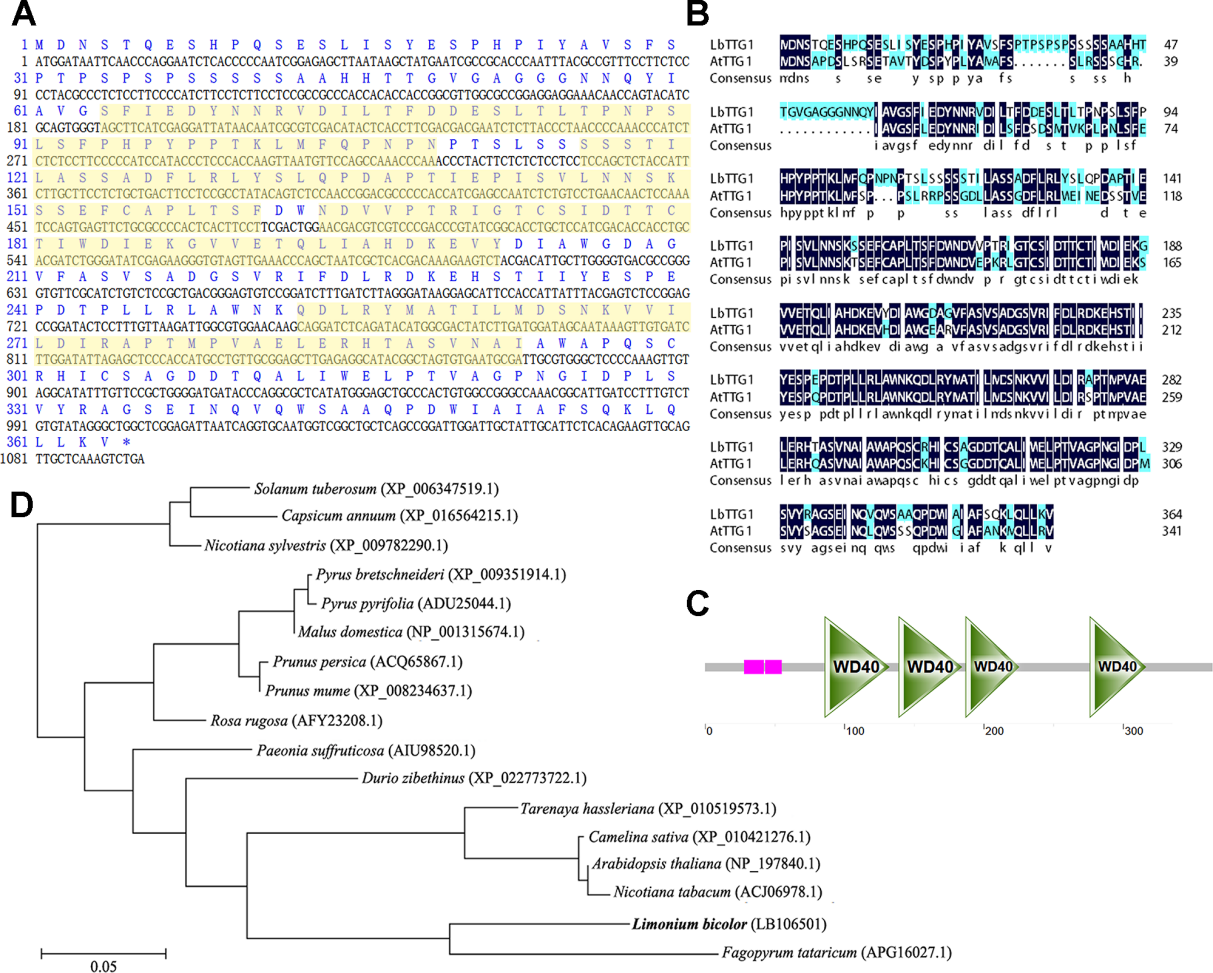
LbTTG1 encoded WD40 repeat protein. **(A)** Nucleotide and amino acid sequences of LbTTG1 analyzed with DNAman. The yellow shadows indicated four WD-repeat domains. **(B)** The sequence alignment of TTG1 between *Limonium bicolor* and *Arabidopsis*. The identity is 73.9%. **(C)** Four conserved WD-repeat domains of LbTTG1, located in 86∼132, 139∼184, 187∼225, and 276∼316 amino acids; pink mean the low complexity domain, drawn with SMART. **(D)** Phylogenetic relationships of different plants based on amino acid residues of LbTTG1, reconstructed by the neighbor-joining method using MEGA and ClustalX software. Bar, 0.05 substitutions per amino acid position.

The *LbTTG1* gene contained a complete ORF of 1,095 bp encoding a protein comprising 364 amino acids with a molecular mass of 39.7 kDa (C_1761_H_2712_N_462_O_561_S_10_) (**Figure 1A**). High identity was illustrated between LbTTG1 and AtTTG1 (**Figure 1B**). LbTTG1 contained four WD-repeat domains located at 86∼132, 139∼184, 187∼225, and 276∼316 amino acids (**Figure 1C**), representing regions highly conserved with other TTG1 proteins. To investigate the evolutionary relationships among TTG1 proteins in plants, we reconstructed a phylogenetic tree using full-length amino acid sequences by the neighbor-joining method. LbTTG1 shared the highest evolutionary relationship with a protein from Tartary buckwheat (*Fagopyrum tataricum*) (**Figure 1D**).

### Subcellular Localization and Expression of LbTTG1 at Different Leaf Developmental Stages

**Figure 2:**
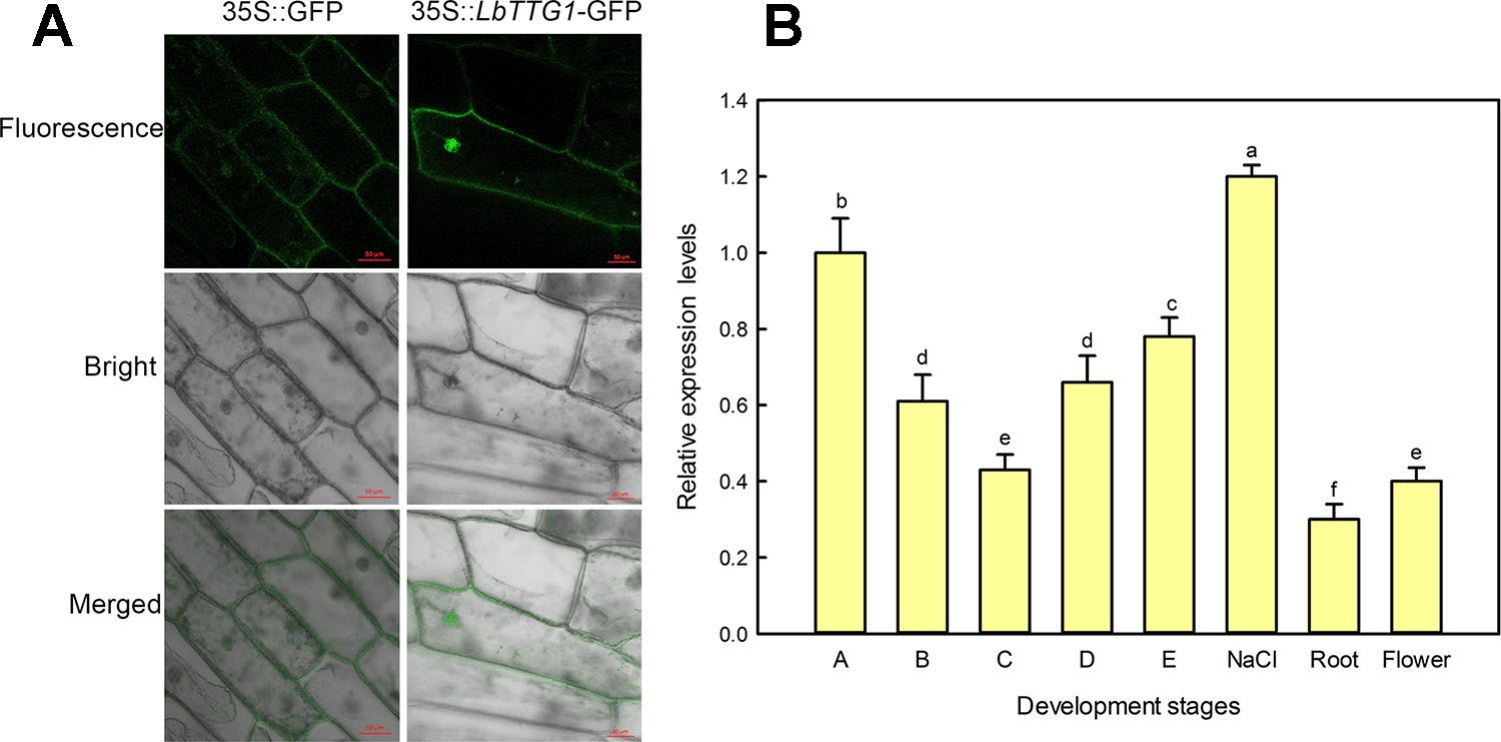
Subcellular localization by onion epidermis transformation and expression level of LbTTG1 at different development stages and conditions. **(A)** Subcellular localization of LbTTG1 after transformation of onion epidermis. GFP-LbTTG1 fusion protein was expressed in both the nucleus and cytoplasm. 35S::GFP is the empty control vector, and 35S::LbTTG1-GFP is the experiment group. Bars = 50 µm. **(B)** Expression levels of *LbTTG1* at different developmental stages and conditions. Stages A-E were the continues leaf development stage. Stage A, undifferentiation, 4–5 days after sowing; stage B, salt gland differentiation, 6–7 days; stage C, stomata differentiation, 8–10 days; stage D, epidermis differentiation, 11–16 days; stage E, mature, ≥17 days after sowing. NaCl means the mature leaves isolated from the seedlings at stage E under 200 mM NaCl for 24 h. Roots were taken from the seedlings of stage E. Flower was the mixture of flower buds and complete flowers.

We transformed onion epidermis cells with *Agrobacterium* containing the p1300-LbTTG1 vector and detected expression of the GFP-LbTTG1 fusion protein in both the nucleus and plasma membrane, while the empty vector only positioned in the membrane (**Figure 2**).

What is the level of *LbTTG1* expression in different organs, different leaf development, and conditions? qPCR was applied to evaluate the effects at different developmental stages and conditions (**Figure 2**). During leaf development (A–E), the highest *LbTTG1* expression levels were identified at stage A and the lowest at stage C (**Figure 2**). The highest expression of *LbTTG1* is showed under 200 mM NaCl treatment. Basal expression level was observed in roots and flowers.

### LbTTG1 Participated in Epidermis Development

**Figure 3:**
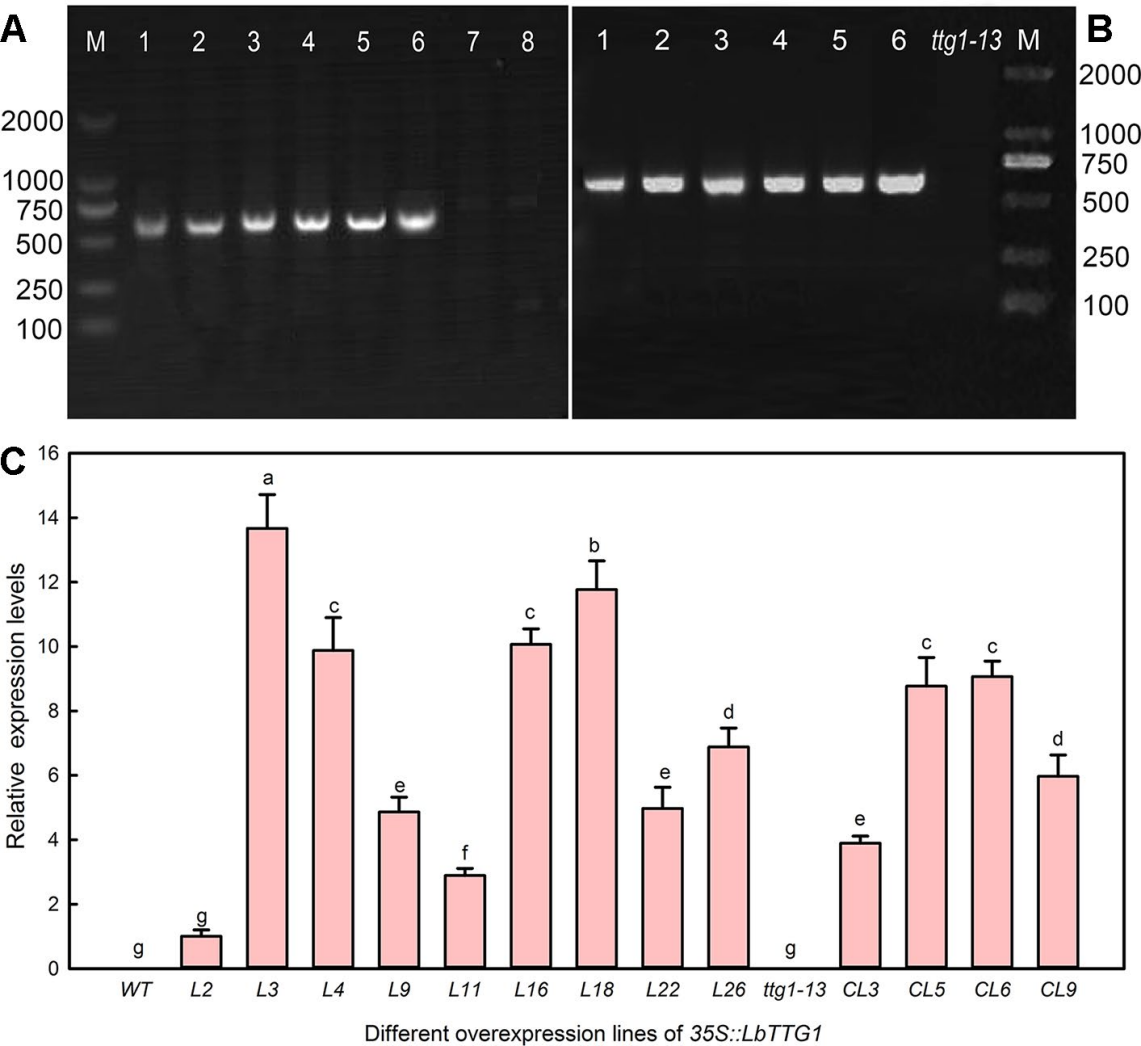
Identification of *Arabidopsis* lines heterologously expressing *LbTTG1*. **(A)** Genomic DNA PCR of *Col* 35S::*LbTTG1* lines; lanes 1–6, different transgenic lines; lane 7, blank control with ddH_2_O as template; lane 8, negative control with wild type DNA as template. **(B)** Genomic DNA PCR of *ttg* 35S::*LbTTG1* lines (lanes 1–6). **(C)** Expression levels of *LbTTG1* examined using quantitative PCR in *Col* 35S::*LbTTG1* and *ttg* 35S::*LbTTG1*. L numbers represent lines of *Col* 35S::*LbTTG1*, e.g. L2 and L3; CL numbers represent complementation lines of *ttg* 35S::*LbTTG1*. Data are means of three replicates ± SD; different letters indicate significant differences at *P* = 0.05 according to Duncan’s multiple range test.

To clarify the relationship between *LbTTG1* and *AtTTG1* and their roles in trichome formation, we heterologously expressed *LbTTG1* in Col-0 (**Figure 3A**) and *ttg1-13* (**Figure 3B**). We identified nine transgenic *Col* 35S::*LbTTG1* lines with high expression of *LbTTG1*, L2, L3, L4, L9, L11, L16, L18, L22, and L26, whereas there was no expression in wild type plants (**Figure 3C**). Among the *ttg* 35S::*LbTTG1* lines, distinctly high expression levels were observed in CL3, CL5, CL6, and CL9, whereas the mutant *ttg1-13* showed no *LbTTG1* expression (**Figure 3C**).

**Figure 4:**
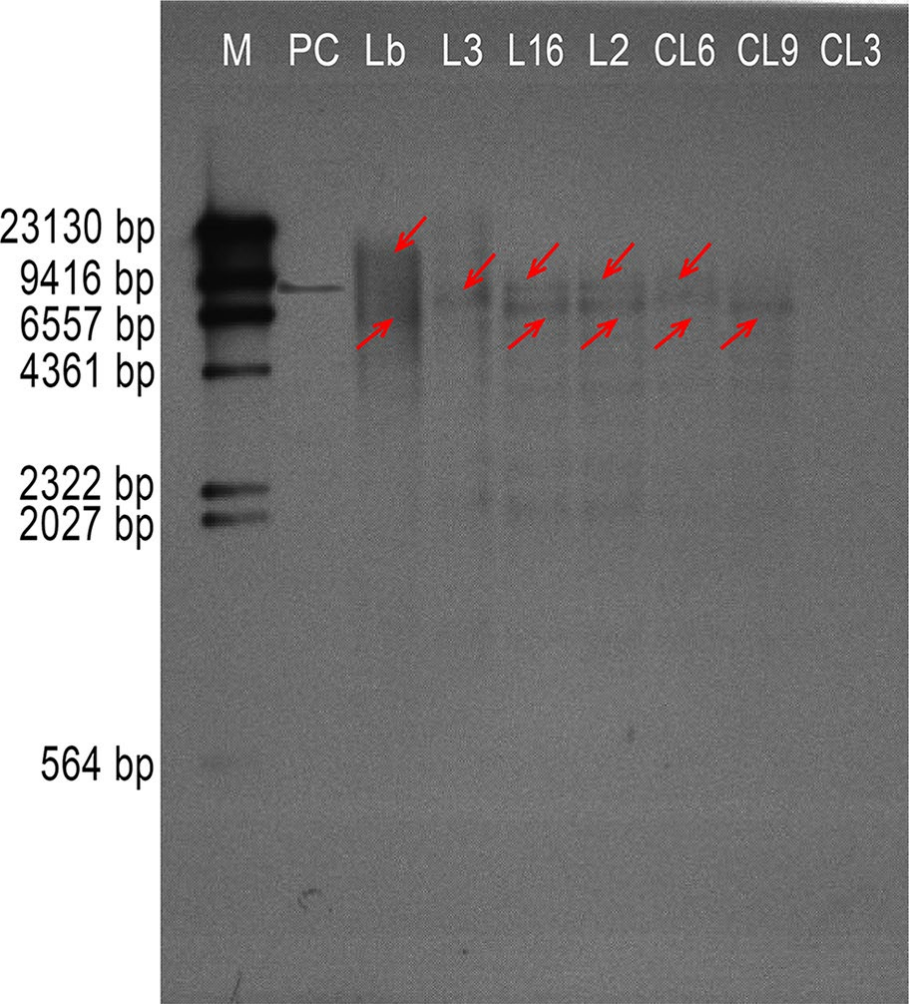
Southern hybridization of *LbTTG1* in *Limonium bicolor* and transgenic lines of *Arabidopsis*. Lane M, DIG-labeled DNA molecular-weight marker; lane PC, positive control; lane Lb, genomic from *L. bicolor*; lane L3, L16, and L2, *Arabidopsis Col* 35S::LbTTG1; lane CL6, CL9, and CL3, *Arabidopsis ttg* 35S::LbTTG1. The red arrow indicated the possible gene copy.

AtTTG1 participates in development of the epidermis, including trichomes and root hairs. We therefore chose three lines harboring each transgene construct with high, medium, and low expression, respectively (L3, L16, and L2 of *Col* 35S::*LbTTG1* and CL6, CL9, and CL3 of *ttg* 35S::*LbTTG1*), to compare the effect of the heterologous expression of *LbTTG1* on the development of trichomes and root hairs. In order to determine the copies of *LbTTG1* in the *L. bicolor* and transgenic *Arabidopsis* genome, Southern blotting was first investigated (**Figure 4**). Two hybridization bands were detected in the genomic of *L. bicolor*. In transgenic lines of *Arabidopsis*, L3 of *Col* 35S::LbTTG1 showed single copy insertion though expression was the highest. L16, L2 of *Col* 35S::LbTTG1, and CL6, CL9 of *ttg* 35S::LbTTG1, exhibited two copies. The above Southern blot analysis confirmed that L3, L16, L2, CL6, and CL9 were stably transformed with *LbTTG1*. However, no bands were detected in line CL3 by southern blotting while PCR verification was positive (**Figure 3**) for LbTTG1 gene, which may be not stable in transgenic *Arabidopsis*.

**Figure 5:**
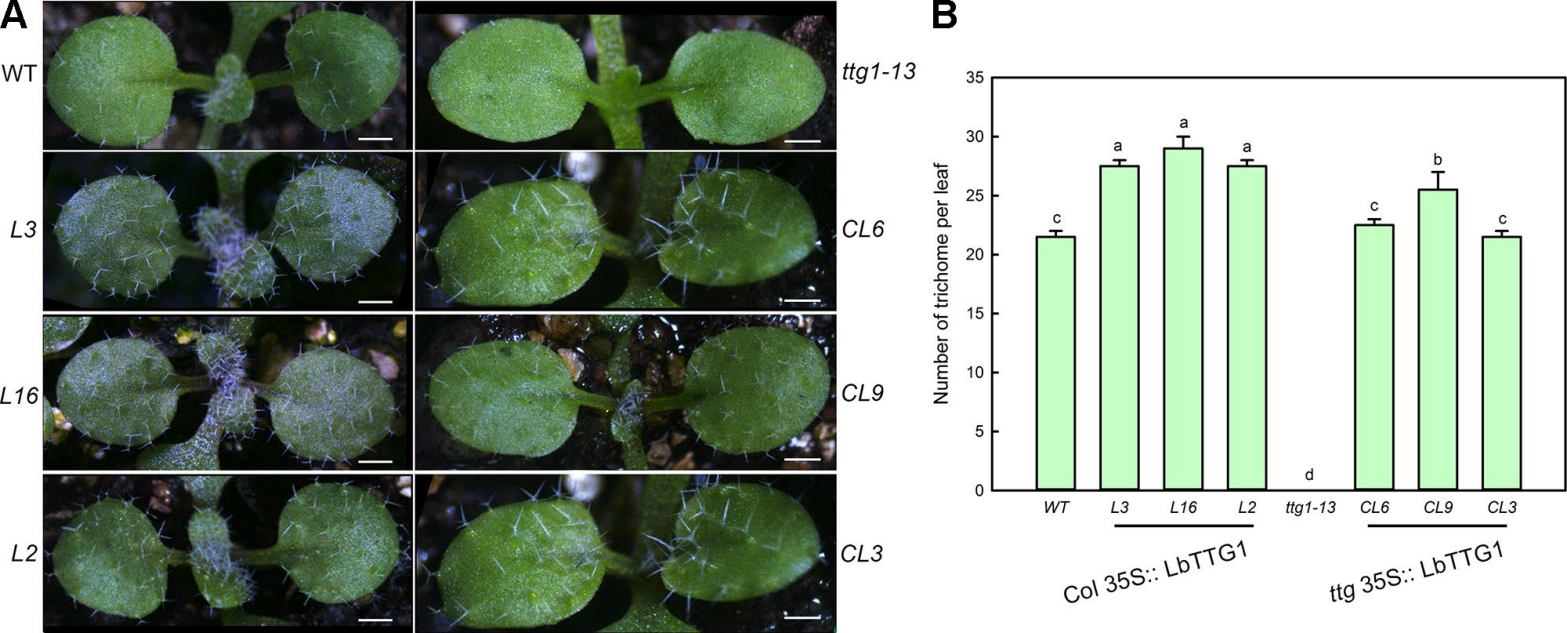
Trichome development was enhanced after transformation of *LbTTG1*. **(A)** Trichomes on the first two rosette leaves of Col-0 wild type (WT), *ttg1-13*, *Col* 35S::LbTTG1 (L3, L16, and L2), and *ttg* 35S::LbTTG1 (CL6, CL9, and CL3). Photographs show 2-week-old soil-grown seedlings. **(B)** Trichome density on the first two rosette leaves of WT, *ttg1-13*, *Col* 35S::*LbTTG1*, and *ttg* 35S::*LbTTG1*. Data are mean ± SD of 10 plants; different letters indicate significant differences at *P* = 0.05 according to Duncan’s multiple range test.

We compared the number of trichomes per leaf in wild type, *ttg 1-13*, *Col* 35S::*LbTTG1*, and *ttg* 35S::*LbTTG1* plants (**Figure 5**). Lines heterologously expressing *LbTTG1* in a wild type background showed enhanced trichome formation; however, the expression level of *LbTTG1* did not have a dose effect on trichome formation. The *ttg1-13* mutant showed no trichome development, while heterologous expression of *LbTTG1* in these mutants returned trichome number to wild type levels. Line CL9 showed more trichome differentiation than the wild type; however, no significant expression level dose effect was observed. In addition, heterologous expression of *LbTTG1* had no effect on the number of trichome branches.

**Figure 6:**
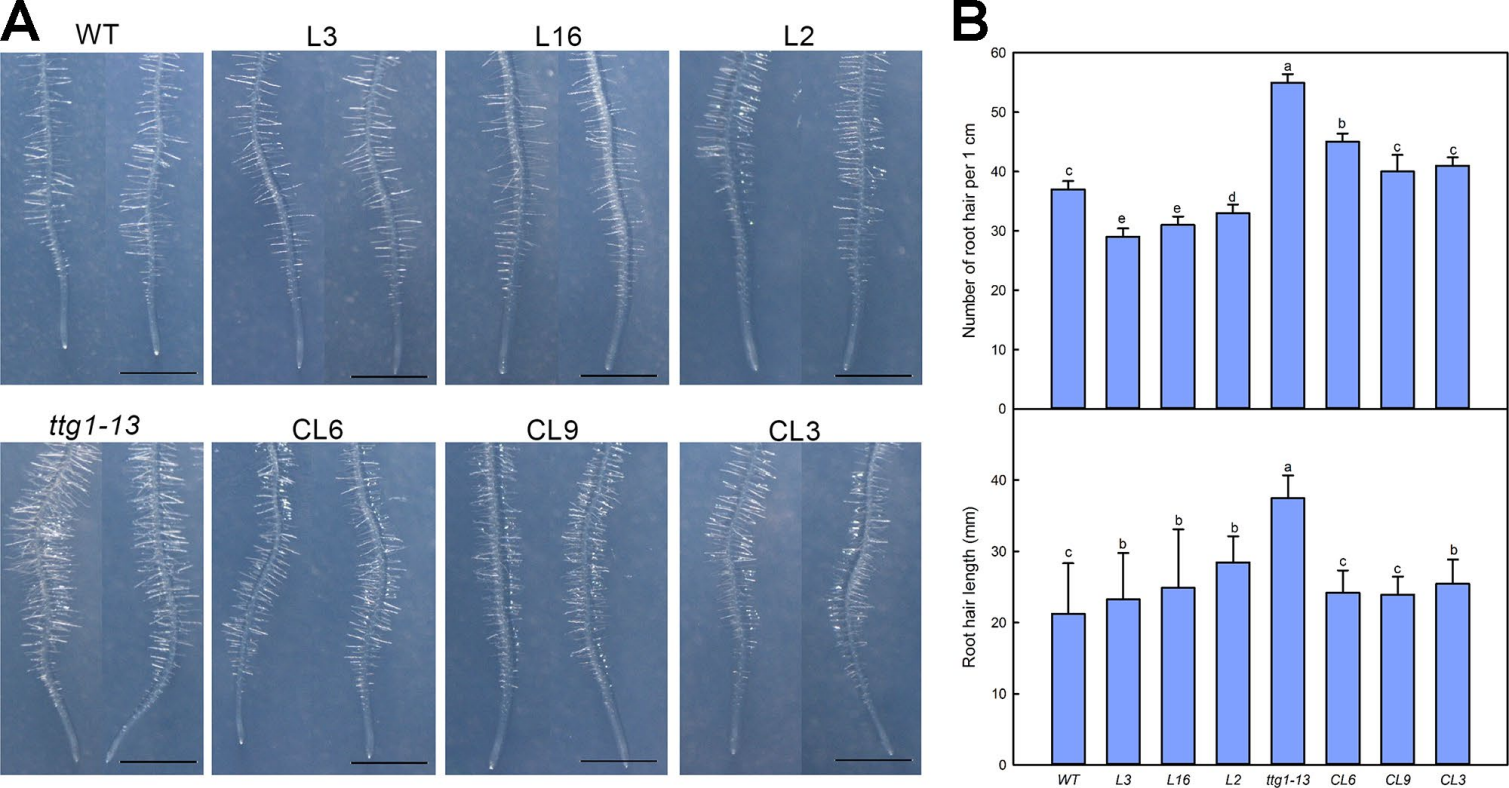
Root hair development of *Col* 35S::*LbTTG1* and *ttg* 35S::*LbTTG1*. **(A)** Phenotypes of root hairs of WT, *ttg1-13*, *Col* 35S::*LbTTG1* (L3, L16, and L2), and *ttg* 35S::*LbTTG1* (CL6, CL9, and CL3) after culture for 5 days on MS medium. **(B)** The same position of each root (region from 2 cm to 3 cm from root tip) was chosen for calculating root hair number and length from 10 or 20 plants from each line. Data for root hair number are mean ± SD of 10 plants. Data for root hair length are mean ± SD of 20 plants; different letters indicate significant differences at *P* = 0.05 according to Duncan’s multiple range test.

We also measured the development of root hairs in the *LbTTG1* expression lines and complementation lines (**Figure 6**). The TTG1 mutant *ttg1-13* had considerably more root hairs than the wild type, whereas all *Col* 35S::*LbTTG1* lines (L3, L16, L2) had fewer. The numbers of root hairs in CL6, CL9, and CL3 *ttg* 35S::*LbTTG1* plants were similar to that in the control (**Figure 6**). Root hair length showed a similar trend; root hairs were longest in *ttg1-13* plants and similar in the complementation lines and wild type. Root hair development was significantly affected by heterologous expression of LbTTG1 in both the wild type and *ttg1-13* mutant.

### Transgenic Lines of LbTTG1 Behave Well in Both Germination Stage and Seedling Stage Under NaCl Treatment

**Figure 7:**
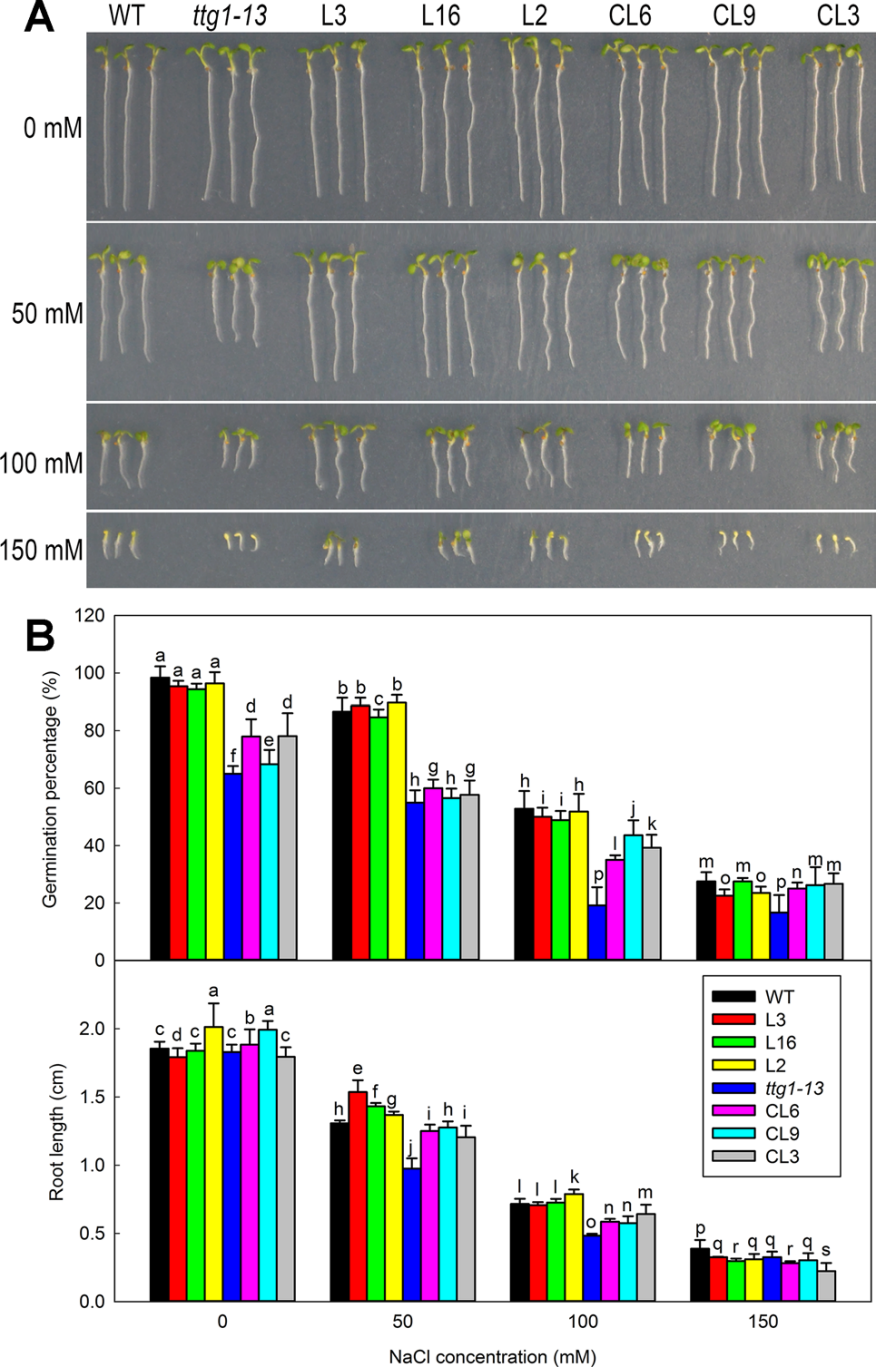
Germination of *Col* 35S::*LbTTG1* and *ttg* 35S::*LbTTG1* seeds under different NaCl concentrations. **(A)** Uniform, 1-day-old seedlings were transplanted to medium containing different NaCl concentrations (0, 50, 100, and 150 mM). Phenotypes of WT, *ttg1-13*, *Col* 35S::*LbTTG1* (L3, L16, and L2), and *ttg* 35S::*LbTTG1* (CL6, CL9, and CL3) in 5-day-old seedlings. **(B)** Germination percentage calculated 24 h after sowing on medium containing different NaCl concentrations (0, 50, 100, and 150 mM). Fifty seeds of each line were sowed in each treatment, and three replicates were performed. Data for germination percentage are mean ± SD. Root length of five-day-old seedlings was calculated using ImageJ software. Data for root length are mean ± SD of 20 plants per line; different letters indicate significant differences at *P* = 0.05 according to Duncan’s multiple range test.

Given that root hairs of transgenic lines were significantly affected, seed germination was further measured using different lines of *Col* 35S::*LbTTG1* and *ttg* 35S::*LbTTG1* (**Figure 7**). Seeds of different lines were treated with different concentrations of NaCl (0, 50, 100, and 150 mM) to analyze the effect of NaCl on germination at 5 days after sowing (**Figure 7**). With increasing NaCl concentration, seeds of all lines showed decreased germination. The *Col* 35S::*LbTTG1* lines (L3, L16, L2) showed similar germination trends to the wild type. The mutant *ttg1-13* showed much poorer germination than the wild type under all treatments, while the *ttg* 35S::*LbTTG1* complementation lines (CL6, CL9, CL3) showed higher germination than *ttg1-13* but still poorer germination than the wild type. Root length showed similar trends under the different NaCl treatments (**Figures 7A, B**).

**Figure 8:**
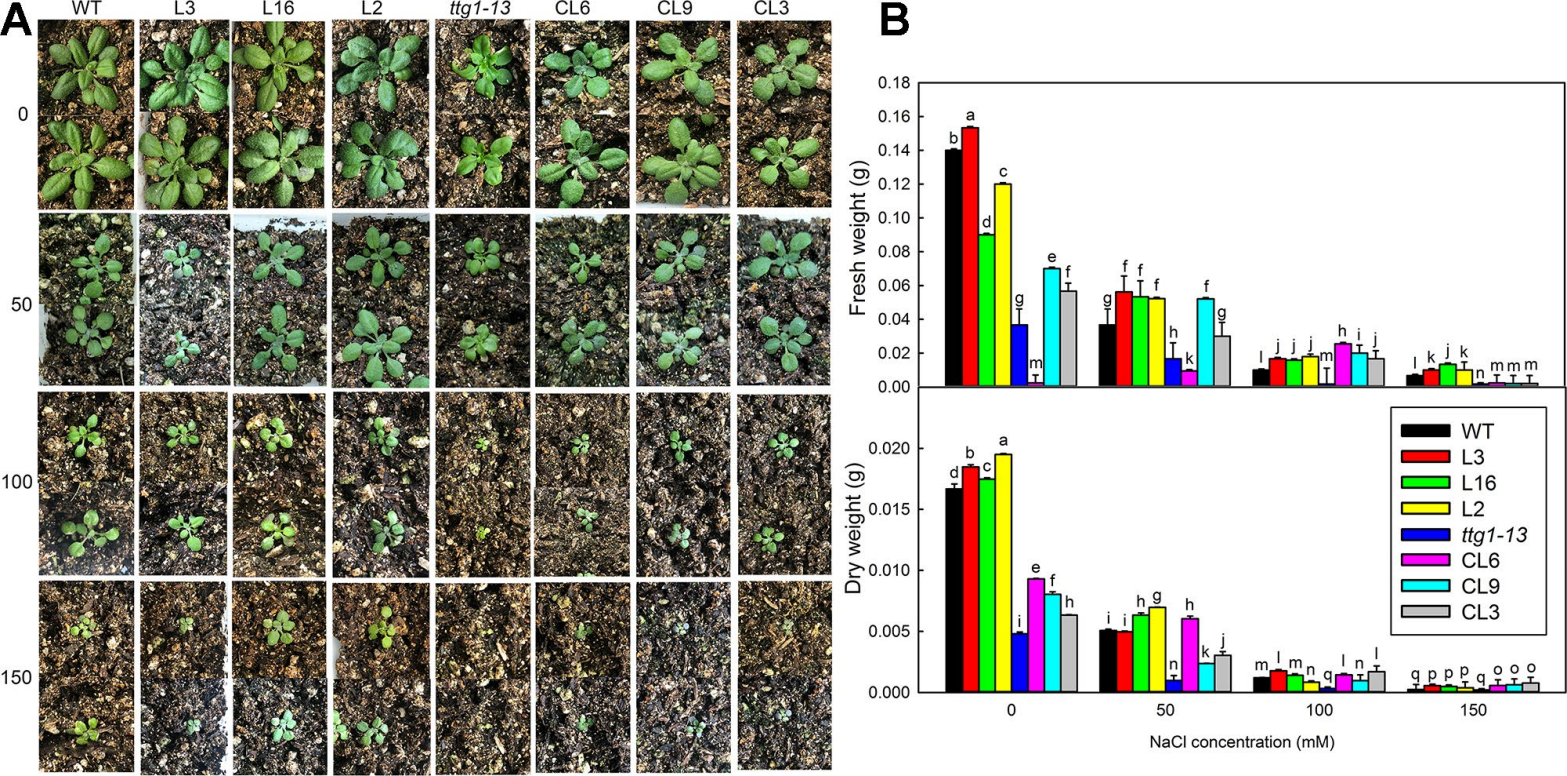
The growth and biomass of different strains under different NaCl treatments. **(A)** Two-week-old seedlings of WT, *ttg1-13*, *Col* 35S::*LbTTG1* (L3, L16, and L2), and *ttg* 35S::*LbTTG1* (CL6, CL9, and CL3) under different NaCl treatments (0, 50, 100, and 150 mM). **(B)** Fresh weight and dry weight of intact seedlings under different NaCl treatments. Data are means ± SD of five replicates; different letters indicate significant differences at *P* = 0.05 according to Duncan’s multiple range test.

**Figure 9:**
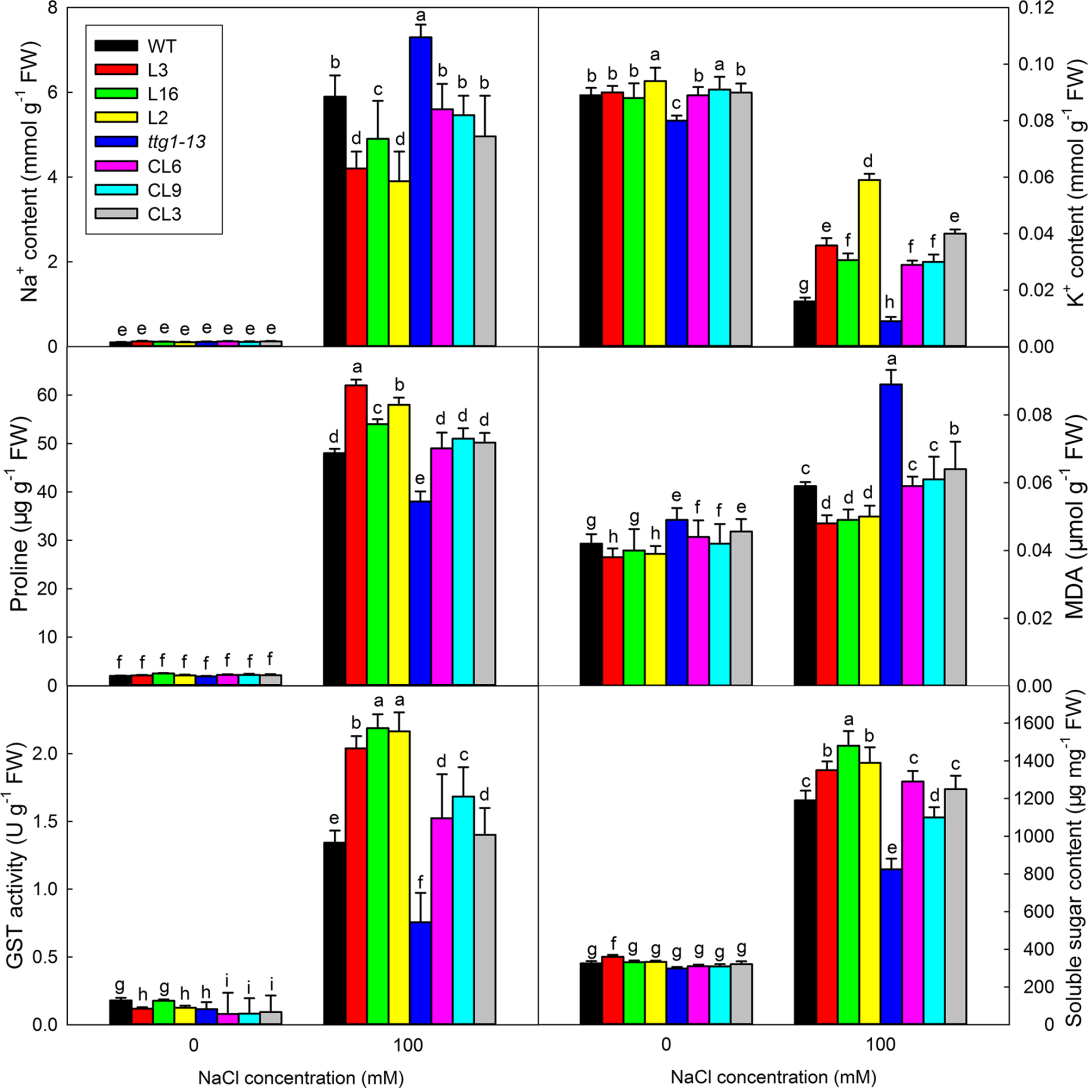
Ion contents, proline, MDA, GST activity, and soluble sugar contents of seedlings under different NaCl treatments. Seedlings under 0 and 100 mM NaCl treatments were pooled for measurement of Na^+^ and K^+^ contents, proline, MDA, GST activity, and soluble sugar contents. Data are means ± SD of five replicates; different letters indicate significant differences at *P* = 0.05 according to Duncan’s multiple range test.

There was no significant difference in the growth of all lines under no-salt conditions, but growth of *ttg1-13* showed a distinct decrease with increasing NaCl concentration (**Figure 8**) and no true leaf was seen under 150 mM treatment. Different from *ttg1-13*, seedlings of *Col* 35S::*LbTTG1* and *ttg* 35S::*LbTTG1* performed better, with L16 and L2 especially producing greater biomass than the wild type under 150 mM NaCl (**Figure 8**). This indicated that the LbTTG1 transgenic lines had enhanced salt tolerance. Better growth (**Figure 8**) and increased biomass (**Figure 8**) of complementation lines compared with *ttg1-13* also supported this conclusion. Given that 100 mM NaCl treatment dramatically inhibited the growth and biomass of all lines, we chose to use the 0 and 100 mM NaCl conditions for further in-depth measurement of physiological indicators, including Na^+^ and K^+^, proline, MDA, GST activity, and soluble sugar (**Figure 9**). No distinct differences were observed among different lines under control conditions; however, under 100 mM NaCl treatment, *ttg1-13* showed the highest Na^+^ content and MDA content and the lowest K^+^, proline, GST activity, and soluble sugar contents. By contrast, the transgenic lines had the lowest Na^+^ content and MDA content and the highest K^+^, proline, GST activity, and soluble sugar contents. These results indicated that the highly salt-tolerant transgenic lines benefited from accumulation of high levels of organic osmotic adjustment substances, low ion toxicity, and high GST activity.

### Expression Levels of Epidermis Differentiation and Stress-Related Marker Genes

**Figure 10:**
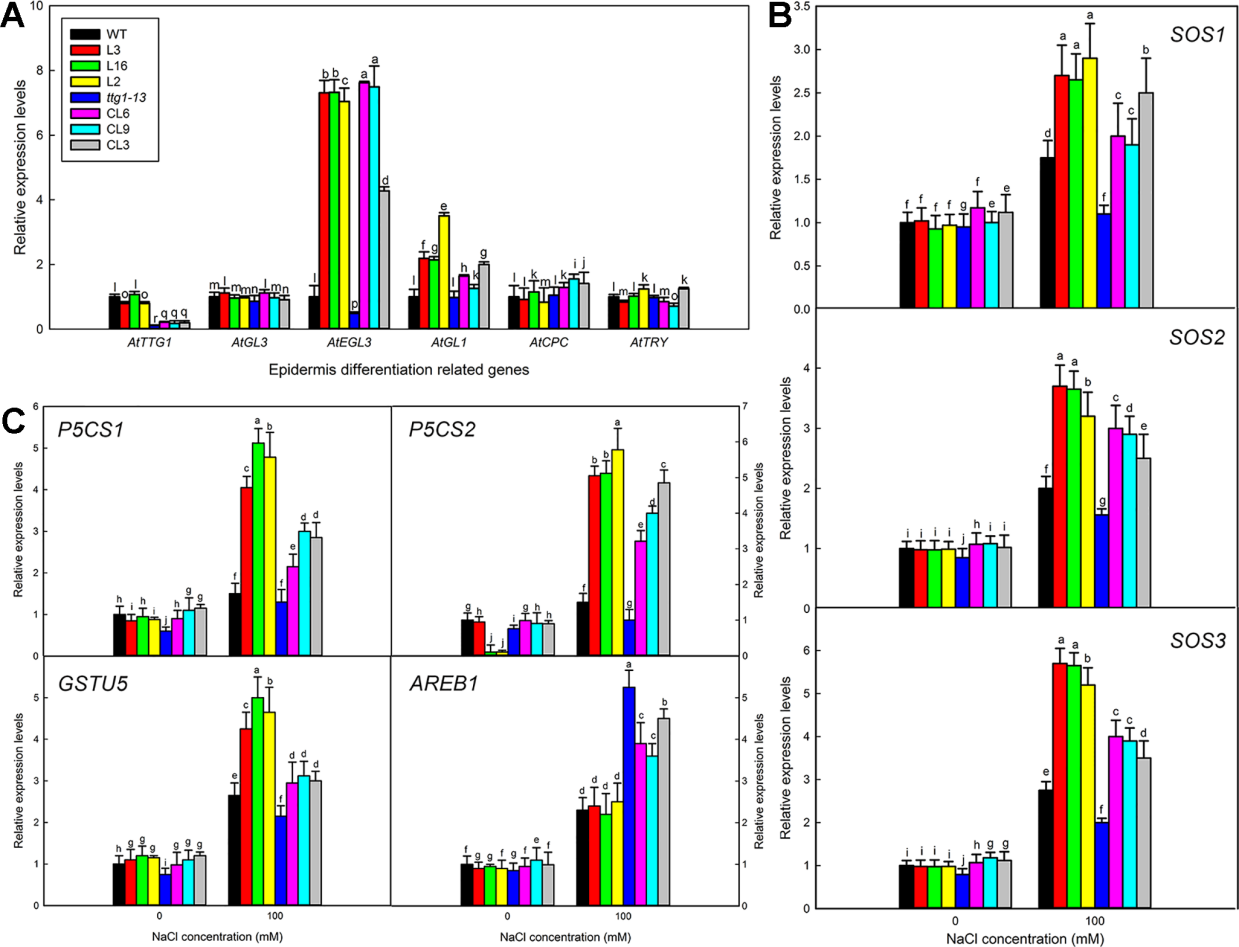
Expression levels of trichome formation and stress-related marker genes. **(A)** Relative expression levels of *AtTTG1*, *AtGL3*, *AtEGL3*, *AtGL1*, *AtCPC*, and *AtTRY* in 5-day-old seedlings. **(B)** Relative expression levels of *AtSOS1*, *AtSOS2*, *AtSOS3*, *AtAREB1*, *AtP5CS1*, *AtP5CS2*, and *AtGSTU5*. Two-week-old seedlings of all lines germinated on MS medium were transferred to 1/2 MS liquid medium containing 100 mM NaCl for 0 and 3 h. Expression of each gene was measured in three replicate biological experiments; different letters indicate significant differences at *P* = 0.05 according to Duncan’s multiple range test.

Transformation of LbTTG1 conferred high heterologous expression levels of LbTTG1 in *Col* 35S::*LbTTG1* and *ttg* 35S::*LbTTG1* plants (**Figure 3**). Considering that trichome differentiation was enhanced (**Figure 5**) and root hair development was inhibited (**Figure 6**) in transgenic lines, we examined key genes involved in epidermis differentiation: *AtTTG1*, *AtGL3*, *AtEGL3*, *AtGL1*, *AtCPC*, and *AtTRY* (**Figure 10**). LbTTG1 transformation did not alter the expression of *AtTTG1* from that of the wild type or *ttg1-13*. No significant differences were observed in the expression of *AtGL3*, *AtCPC*, or *AtTRY* among different lines. Two genes (*AtEGL3* and *AtGL1*) showed their highest expression in transgenic lines; these proteins may directly combine with LbTTG1 to promote trichome development and inhibit root hair differentiation.

To investigate why transformation of LbTTG1 significantly improved the salt tolerance of *Arabidopsis*, we chose seven stress-related marker genes for preliminary investigation (**Figures 10B, C**): *AtSOS1* (Salt overly sensitive 1), *AtSOS2* (Salt overly sensitive 2), *AtSOS3* (Salt overly sensitive 3), *AtP5CS1* (Δ1-pyrroline-5-carboxylate synthetase 1), *AtP5CS2* (Δ1-pyrroline-5-carboxylate synthetase 2), *AtGSTU5* (GST class tau 5), and *AtAREB1* (abscisic acid-responsive element binding protein 1). Under short-term 100 mM NaCl treatment, high expression levels of *AtSOS1*, *AtSOS2*, *AtSOS3*, *AtP5CS1*, *AtP5CS2*, and *AtGSTU5* were seen in the transgenic expression lines in the wild type background (L3, L16, and L2), while the lowest expression levels occurred in *ttg1-13*, and expression levels in the complementation lines (CL6, CL9, and CL3) were intermediate between the two (**Figures 10B, C**). A different trend in expression was seen for the abscisic acid-related gene *AREB1*, with highest expression in *ttg1-13* and relatively lower expression in transgenic lines (**Figure 10C**).

## Discussion

LbTTG1 of *L. bicolor* contains the similar WD40-repeat protein domains as TTG1 proteins from other species (Brueggemann et al., 2010), which may determine epidermis differentiation into trichomes and root hairs (Schellmann and Hulskamp, 2004). Heterologous expression of *LbTTG1* in *Arabidopsis* increased trichome number (**Figure 5**) and decreased root hair number (**Figure 6**). Moreover, we observed no dose effect of *LbTTG1* expression on trichome and root hair development, indicating that LbTTG1 can enhance the function of AtTTG1 in the positive regulation of trichome formation and negative regulation of root hair development, and a low level of LbTTG1 is sufficient for the formation of the TTG1-EGL3-GL1 complex with EGL3 and GL1 to initiate trichome and root hair differentiation. We used AtTTG1 deletion mutants of *Arabidopsis* for the heterologous expression of LbTTG1 to illustrate the homology between LbTTG1 and AtTTG1 in epidermis differentiation. Although different complementation lines showed different LbTTG1 expression levels and gene copies, we observed distinct complementation phenotypes similar to those of the wild type, with more trichomes (**Figure 5**) and fewer root hairs (**Figure 6**) than in the mutant. All these results indicate that, similar to TTG1 proteins from other species, LbTTG1 and AtTTG1 have homologous functions in epidermis differentiation, probably due to their common WD40 domain (Ben-Simhon et al., 2011; Schaart et al., 2013; Dressel and Hemleben, 2009).

Besides *Arabidopsis*, the function of TTG1 protein is well illustrated in *Setaria italica*, *Artemisia annua*, and others. Similar to what we observed here, SiTTG1 of *S. italica* (Liu et al., 2017) and AaWD40 from *A. annua* (Wang et al., 2016) can complement the trichome phenotype of *ttg1-13* to restore the wild type phenotype of *Arabidopsis*, and the latter can enhance the synthesis of anthocyanin. Similar results of enhanced trichome formation were seen in transgenic lines of CsWD40 from *Camellia sinensis* (Liu et al., 2018). *TTG1* genes in different species thus show high conservation in controlling epidermis differentiation. The WD40-repeat domain may be responsible for this, but further transformation with WD40-repeat domain deletions of LbTTG1 will allow deeper understanding of the mechanism. How does LbTTG1 participate in epidermis differentiation? Two key genes (*AtEGL3* and *AtGL1*) involved in epidermis development were highly expressed in transgenic lines (**Figure 10**). (Zhao et al., 2008) reported that AtTTG1 combines with EGL3 and GL1 to initiate trichome formation. The current study using heterologous expression in *Arabidopsis* suggested that LbTTG1 may also combine with EGL3 and GL1 to promote trichome formation and inhibit root hair differentiation. Transcriptome and *in vitro* interaction analyses will provide details of how the downstream and interacting proteins function in the future.

In addition to its function in epidermis differentiation, which is similar to that of its homologs in other species, a specific function of salt tolerance was seen in LbTTG1 transgenic lines in both wild type and *ttg1-13* backgrounds. Interestingly, *ttg1-13* showed sensitivity to salt, which may be due to increased root hair development (**Figure 6**), whereas the complementation lines showed increased germination over that of *ttg1-13* under increasing NaCl treatment (**Figure 7**). Similar trends were seen in physiological indicators, including MDA and ion contents. This may be explained in two ways. On the one hand, we can obtain clues from ion contents under different treatments (**Figure 9**). More root hairs lead to more Na^+^ being absorbed into the root, so the *ttg1-13* mutant showed salt sensitivity due to accumulated Na^+^ and ionic homeostasis disorder; transgenic lines had fewer root hairs, so these plants showed salt tolerance. On the other hand, in addition to the WD40-repeat domain common to all TTG1 proteins, LbTTG1 was also speculated to have a specific domain (maybe the low complexity domain in **Figure 1C**) that may determine the specific function of salt tolerance in this halophyte, which had highest expression level under NaCl treatment (**Figure 2**). We examined expression levels of marker genes for salt tolerance in LbTTG1 transgenic lines (**Figures 10B, C**), including Na^+^ efflux, osmotic adjustment, active oxygen scavenging system, and abscisic acid synthesis. *SOS1*, *SOS2*, and *SOS3* was up-regulated in transgenic lines (**Figure 10**); *SOS3* showed the most significant increase among the three, which may combine with SOS2, and then active SOS1 (a Na^+^/H^+^ antiporter located in the plasma membrane) to actively transport Na^+^ to the outside of the cell, reducing harm to protoplasts (Shi et al., 2000; Qiu et al., 2002; Zhu, 2002). Increased proline accumulation in transgenic lines compared with the wild type and *ttg1-13* (**Figure 9**) may be due to high expression levels of *AtP5CS1* and *AtP5CS2* (**Figure 10**), which both controlled the synthesis of proline (Mattioli et al., 2010). Organic osmolytes such as proline are synthesized for osmotic adjustment under salt stress (Székely, 2004), so transgenic lines of LbTTG1 performed well under NaCl treatment. In addition to ion stress and osmotic stress, oxidative stress also causes damage under salt stress conditions (Khan and Ashraf, 2008). GST, one of the indicators of antioxidant enzyme system activity (Wagner et al., 2002), showed high expression in transgenic lines (**Figure 9**), which was likely due to the high expression level of *AtGSTU5* (**Figure 10**), thus enhanced the salt tolerance of the transgenic lines. Salt stress often induces synthesis of abscisic acid and up-regulation of AREB1 (Fujita et al., 2005; Narusaka et al., 2003). Under salt treatment, *ttg1-13* showed higher *AREB1* expression levels than transgenic lines, which might also explain the high salt tolerance of transgenic lines.

In summary, transgenic lines of *Arabidopsis* heterologously expressing *LbTTG1* showed improved salt tolerance at both the germination and seedling stages. The epidermis of transformed plants displayed enhanced trichome and reduced root hair development. A specific domain of LbTTG1 and the common WD40-repeat domain may be responsible for these phenotypes. Given that a transformation system is available for *L. bicolor*, further studies using the CRISPR-cas9 system will be performed to verify the function of *LbTTG1*.

## Data Availability Statement

The raw data supporting the conclusions of this manuscript will be made available by the authors, without undue reservation, to any qualified researcher.

## Author Contributions

FY and BW designed research. BL, HZ, and XW performed research. BL and GH analyzed data. FY wrote the paper. BW revised the paper.

## Funding

This work was supported by the NSFC (National Natural Science Research Foundation of China, project nos. 31600200; 31570251; 31770288), Shandong Province key research and development plan (2015ZDJS03002; 2017CXGC0313), the Natural Science Research Foundation of Shandong Province (ZR2014CZ002), and the Higher Educational Science and Technology Program of Shandong Province (J15LE08).

## Conflict of Interest

The authors declare that the research was conducted in the absence of any commercial or financial relationships that could be construed as a potential conflict of interest.
