# Supplementary material for: A WD40-Repeat Protein From the Recretohalophyte Limonium bicolor Enhances Trichome Formation and Salt Tolerance in Arabidopsis
Source: Front Plant Sci. 2019 Nov 12;10:1456. doi: 10.3389/fpls.2019.01456 (PMC6861380; doi:10.3389/fpls.2019.01456)
Supplement: Supplementary file 1 [file Table_1.docx]

Table S1 The primers used in the current paper.

| **Name** | **Oligonucleotide sequence** | **Role** |
| --- | --- | --- |
| *LbTTG1*-S | 5-GTATCGGCACCTGCTCCATC-3 | Amplification of specific fragment |
| *LbTTG1*-A | 5-ACACTAGCCGTATGCCTCTC-3 |  |
| *LbTTG1*3’-GSP1 | 5-GAGCATTCCACCATTATTTACGAGTC-3 | 3’RACE |
| *LbTTG1*3’-GSP2 | 5-AAGATTGGCGTGGAACAAGCAG-3 |  |
| QT | 5-CCAGTGAGCAGAGTGACGAGGACTCGAGCTCAAGCTTTTTTTTTTTTTTTTT-3 |  |
| Q0 | 5-CCAGTGAGCAGAGTGACG-3 |  |
| Q1 | 5-GAGGACTCGAGCTCAAGC-3 |  |
| *LbTTG1*5’-GSP1 | 5-TGCTTGTTCCACGCCAATCTTA-3 | 5’RACE |
| *LbTTG1*5’-GSP2 | 5-GGAGACTCGTAAATAATGGTGG-3 |  |
| *LbTTG1* CDS-S | 5-ATGGATAATTCAACCCAGGAAT-3 | Full length amplification |
| *LbTTG1* CDS-A | 5-TCAGACTTTGAGCAACTGCAAC-3 |  |
| *LbTTG1* OE-S | 5-CGGGGTACCCCGATGGATAATTCAACCCAGGAATCT-3 | Construction of pCAMBIA1300 -LbTTG1 vectors digested with KpnⅠand BamHⅠ |
| *LbTTG1* OE-A | 5-CGCGGATCCGCGGACTTTGAGCAACTGCAACT-3 |  |
| *LbTTG1* RT-S | 5-ATGATACACAGGCTCTTA-3 | Real-time PCR in different developmental stages and Arabidopsis overexpression lines |
| *LbTTG1* RT-A | 5-AAGCAATACCAATCCAAT-3 |  |
| *Lbtubulin* RT-S | 5-GGTTGAGTGAGCAGTTCAC-3 |  |
| *Lbtubulin* RT-A | 5-GATAACCAGCCACACCTTAGC-3 |  |
| *Atactin* RT-S | 5-GGTAACATTGTGCTCAGTGGTGG-3 |  |
| *Atactin* RT-A | 5-AACGACCTTAATCTTCATGCTGC-3 |  |
| *LbTTG1* OEAt-S | 5-GGACTCTTGACCATGGATAATTCAACCCAGGAATCT-3 | Construction of p35S::LbTTG1 |
| *LbTTG1* OEAt-A | 5-CTCAGATCTACCATGGTGACTTTGAGCAACTGCAACT-3 |  |
| *LbTTG1*V-S | 5-AAGATTGGCGTGGAACAAGCAG-3 | Verification of Arabidopsis overexpression lines *Col* 35S::*LbTTG1* and *ttg* 35S::*LbTTG1* |
| *LbTTG1*V-A | 5-TTTGATTTCACGGGTTGGGGTTTC-3 |  |
| *LbTTG1*P-S | 5-TTCTCCCCTACGCCCTCT-3 | Probe synthesis of Southern blotting |
| *LbTTG1*P-A | 5-CACGCAATCGCATTCACA-3 |  |
| *AtTTG1*-S | 5-TATTGAGAAGTCTGTTGT-3 | RT-qPCR verification of trichome formation genes in *Col* 35S::*LbTTG1* and *ttg* 35S::*LbTTG1* |
| *AtTTG1*-A | 5-ATTGTAGAATGTTCCTTATC-3 |  |
| *AtGL1*-S | 5-CCTTCTTCTTGTCATCAT-3 |  |
| *AtGL1*-A | 5-ATCATTAGTAGTTGCCATT-3 |  |
| *AtGL3*-S | 5-GCTTAGATGTGCTTGGAGAG-3 |  |
| *AtGL3*-A | 5-GAGGATTGAACCGAATGAGAA-3 |  |
| *AtEGL3*-S | 5-AATCTTCTGGTCTGTCTC-3 |  |
| *AtEGL3*-A | 5-AATCGTCTTCCTTGTCTT-3 |  |
| *AtCPC*-S | 5-TCCGAAGAGGTGAGTAGT-3 |  |
| *AtCPC*-A | 5-ACGAGTTTATACATCCGAGAA-3 |  |
| *AtTRY*-S | 5-CTTCTTCTTCTTGTTCGCTCTA-3 |  |
| *AtTRY*-A | 5-ACGGTCAGTGTTATCCATTAC-3 |  |
| *AtSOS1*-S | 5-TTCATCATCCTCACAATGGCTCTAA-3 | RT-qPCR verification of trichome formation genes in *Col* 35S::*LbTTG1* and *ttg* 35S::*LbTTG1* |
| *AtSOS1*-A | 5-CCCTCATCAAGCATCTCCCAGTA-3 |  |
| *AtSOS2*-S | 5-GAACTCCGAACTATGTAG-3 |  |
| *AtSOS2*-A | 5-TATCCAGCCAATATAACG-3 |  |
| *AtSOS3*-S | 5-AGAGGAAGATAGAGATGTAAGC-3 |  |
| *AtSOS3*-A | 5-ATTATGTATGTGAGATGGAGAGT-3 |  |
| *AtP5CS1*-S | 5-CAAGATGAGATTACATTCG-3 |  |
| *AtP5CS1*-A | 5-GGTTATGATGACAGGAAT-3 |  |
| *AtP5CS2*-S | 5-GGCTTACTATGAGACTATGT-3 |  |
| *AtP5CS2-*A | 5-ACTAAGTTGCTTCCTGAA-3 |  |
| *AtGSTU5*-S | 5-ATGGCTGAGAAAGAAGAAGTGAAGC-3 |  |
| *AtGSTU5*-A | 5-TTAAGAAGATCTCACTCTCTCTGCC-3 |  |
| *AtAREB1*-S | 5-TCACTCTCTCCGTCTCCTTAC-3 |  |
| *AtAREB1*-A | 5-CATTCTCCTTTGCCTTCTCTCT-3 |  |

QT, Q0 and Q1 was referred to ([Sui et al., 2017](#_ENREF_1)).

Red means restriction sites and part vector sequence for vector fusion.

S means *sense*, and A means *antisense*.

**Sui, N., Tian, S., Wang, W., Wang, M., and Fan, H.** (2017). Overexpression of glycerol-3-phosphate acyltransferase from *Suaeda salsa* improves salt tolerance in Arabidopsis. Frontiers in plant science **8,** 1337.
